# The COMMAND trial of cognitive therapy to prevent harmful compliance with command hallucinations: predictors of outcome and mediators of change

**DOI:** 10.1017/S0033291717003488

**Published:** 2017-12-05

**Authors:** Max Birchwood, Graham Dunn, Alan Meaden, Nicholas Tarrier, Shon Lewis, Til Wykes, Linda Davies, Maria Michail, Emmanuelle Peters

**Affiliations:** 1 Mental Health and Wellbeing, Warwick Medical School, University of Warwick, Coventry, UK; 2 Centre for Biostatistics, Institute of Population Health, University of Manchester, Manchester, UK; 3 Faculty of Health Science, Birmingham City University, Birmingham, UK; 4 School of Psychological Science, University of Manchester, Manchester, UK; 5 Division of Psychiatry, School of Medicine, University of Manchester, Manchester, UK; 6 Institute of Psychiatry, Psychology and Neuroscience, Kings College London, London, UK; 7 Centre for Health Economics – Institute of Population Health, University of Manchester, Manchester, UK; 8 School of Psychology, University of Birmingham, Birmingham, UK

**Keywords:** CBT, voice power, hallucinations, psychosis, harm

## Abstract

**Background:**

Acting on harmful command hallucinations is a major clinical concern. Our COMMAND CBT trial approximately halved the rate of harmful compliance (OR = 0.45, 95% CI 0.23–0.88, *p* = 0.021). The focus of the therapy was a single mechanism, the power dimension of voice appraisal, was also significantly reduced. We hypothesised that voice power differential (between voice and voice hearer) was the mediator of the treatment effect.

**Methods:**

The trial sample (*n* = 197) was used. A logistic regression model predicting 18-month compliance was used to identify predictors, and an exploratory principal component analysis (PCA) of baseline variables used as potential predictors (confounders) in their own right. Stata's *paramed* command used to obtain estimates of the direct, indirect and total effects of treatment.

**Results:**

Voice omnipotence was the best predictor although the PCA identified a highly predictive cognitive-affective dimension comprising: voices’ power, childhood trauma, depression and self-harm. In the mediation analysis, the indirect effect of treatment was fully explained by its effect on the hypothesised mediator: voice power differential.

**Conclusion:**

Voice power and treatment allocation were the best predictors of harmful compliance up to 18 months; post-treatment, voice power differential measured at nine months was the mediator of the effect of treatment on compliance at 18 months.

Acting on delusions and commanding voices, remains a major concern for psychiatry with attendant societal and political concern, where members of the public are subject to apparently random acts of violence, even when they are well supported by services. Harm to self is an even greater risk linked to commanding voices (Birchwood *et al.* 2014). This is reflected in national policy documents, for example, the national mental health strategy in England aimed to reduce ‘avoidable harm’ to self or others (UK Department of Health, 2011).. While drug treatment has improved, approaching 50% will continue to experience treatment-resistant symptoms or symptoms arising from non-adherence to drug regimes (Leucht *et al.* 2009). Auditory hallucinations (AH) rank among the most prominent of the treatment-resistant symptoms and among the most distressing and high risks of all are command hallucinations (Upthegrove *et al.* 2016). Command hallucinations are prevalent in people with schizophrenia and related disorders. A review by Shawyer *et al.* (2003) reported a median prevalence rate of 53% with a wide range from 18% to 89%, in a sample of adult psychiatric patients; of these, 48% stipulated harmful or dangerous actions. Among patients in medium secure units, compliance rises to 69% (Rogers *et al.* 2002). This rate appears significantly higher in the forensic population with 83% of voice hearers experiencing command hallucinations with criminal content (Beck-Sander *et al.* 1997). However, the link between the presence of command hallucinations and harm to self or others is not straightforward. In the Macarthur study (Appelbaum *et al.* 2000), no association was reported between the presence of delusions or command hallucinations and violence (GBH, assault and threats with a weapon). It appears to be the content of the individual's thinking and how this reflects the dynamics of their relationship with the supposed persecutor who is commanding them, that is found to be predictive of harm to self and others (Beck-Sander *et al.* 1997; Bucci *et al.* 2013). Our cognitive model of ‘voices’ has clarified that it is not only the level of activity of voices, or indeed their content, that drives affect and behaviour, but the nature of the relationship with the personified voice (Chadwick & Birchwood, 1994; Birchwood *et al.* 2004; Connor & Birchwood, 2013); see review by Mawson *et al.* (2010). We showed that where the voice hearer believes the voice to have malevolent intent, and crucially to have the power to deliver the threat, this can motivate compliance or appeasement behaviour (Birchwood *et al.* 2004). These findings have been independently replicated in a forensic population (Fox *et al.* 2004). This framework informed the development of our targeted cognitive behaviour therapy (CBT) treatment designed to weaken and challenge a single mechanism, beliefs about voices’ power, thus enabling the individual to break free of the need to comply or appease and thereby reduce harmful compliance behaviour and distress (Byrne *et al.* 2006; Meaden *et al.* 2013). The effect of cognitive therapy for command hallucinations (CTCH) was tested in a full multi-centre trial of 197 individuals with a recent history of harmful compliance, involving harm to themselves or others. This group was observed to have a 46% rate of recurrence of harmful compliance within 18 months in the control group compared with 28% of those receiving CTCH [odds ratio = 0.45, 95% confidence interval (CI) 0.23–0.88, *p* = 0.021]. This was accompanied by a reduction in the perceived power of the voice (the estimated treatment effect common to both time points was −0.52 [95% CI −0.849 to −0.185), *p* = 0.002]. No differential impact on other secondary outcomes was observed.

In our main results paper (Birchwood *et al.* 2014) we highlighted two outstanding questions from our protocol (Birchwood *et al.* 2011), of theoretical and clinical importance. First, what are the key baseline predictors of outcome at 18 months? Here we are interested in whether it is voice beliefs, psychosis severity, demographic and historical factors, treatment allocation, or a combination of these, which predict further acts of harmful compliance; our preliminary study found voice power to be the best individual predictor (Bucci *et al.* 2013). Second, while these results were in line with predictions (changes in power and in compliance) we did not establish voice power as the *mediator* of change, as predicted by our model. This paper addresses each of these questions.

## Method

### Trial design

This was a single (rater) blind, prospective, pragmatic randomised controlled trial, using intention to treat comparing CTCH + Treatment as usual (TAU) with TAU alone. The trial recruited eligible participants from UK sites in Birmingham, Leicester, London and Manchester.

The trial was funded by the Medical Research Council (G0500965), was registered (ISRCTN62304114) and received ethical approval from the West Midlands Research Ethics Committee (06/MRE07/71). Full details on the design and trial findings can be found in the trial protocol (Birchwood *et al.* 2011) and primary publication (Birchwood *et al.* 2014).

### Participants

A total of 197 of 242 eligible participants consented to the trial (81%), fulfilling the following criteria: (i) ICD – 10 schizophrenia, schizoaffective (F20, 22, 23, 25, 28, 29), or mood disorders (F32) (WHO, 2009), under care of a clinical team; (ii) age ⩾16; (iii) history of command hallucinations of at least 6 months with recent (<9 months) history of harm to self, others or major social transgressions as a result of the commands; or harmful command hallucinations where the individual is distressed and appeasing the powerful voice and therefore at risk of full compliance. Exclusion criteria included: organic impairment or addictive disorder considered to be the primary diagnosis; insufficient command of the English language. The mean age of the sample was 37.4 (12.1; 16–64) and 113 (57%) male. Harmful compliance included harm to self (*n* = 119), others (42), kill self and/or other (53). All were in receipt of anti-psychotic medication.

### The intervention and treatment as usual

CTCH is designed to weaken and change beliefs about voices’ power, thus enabling the individual to break free of the need to comply or appease and thereby reduce harmful compliance behaviour and distress. Our CTCH treatment protocol was developed by the first author and detailed in our casebook manuals (Byrne *et al.* 2006; Meaden *et al.* 2013). The primary aim of the therapy is to reduce the power differential between voice and voice hearer. The essence of the therapy is to test out the perceived power of the voice by examining evidence for: (a) the voice hearer's perceived lack of control over voice activity, (b) the perceived omniscience of the voice (e.g. the apparent ability of the voice to predict the future) and (c) the perceived capacity of the voice to carry out its threats for non-compliance. CTCH was administered over a maximum period of 9 months. This includes a therapeutic window up to 25 sessions. TAU was provided by Community Mental Health Teams, Assertive Outreach and Early Intervention Teams. TAU including anti-psychotic medication was documented and did not differ between the groups.

### Measures

#### Compliance

The level of compliance/resistance was assessed using the Voice Compliance Scale (VCS) (Addington *et al.* 1993) after: (a) conducting a thorough interview using the Cognitive Assessment of Voices schedule in order to obtain a detailed record of all voices as well as emotional and behavioural responses towards these voices; (b) interviewing and using information from *at least* one other informant (carers, care-coordinator). This yields specific behaviours which are classified as: neither appeasement nor compliant (1); symbolic appeasement, i.e. compliant with innocuous and/or harmless commands (2); actual appeasement, i.e. preparatory acts or gestures (3); partial compliance with at least one severe command (4); full compliance with at least one severe command (5). Twenty-nine of these were independently rated by MM for the presence *v.* absence of full compliance, with an overall Kappa of 0.73.

The primary outcome was the presence of full compliance (score 5) over the preceding nine months, with documented and independently verified behaviours. This was accordingly treated as a binary measure in the regression analyses.

### Beliefs about voices

#### Power

The Voice Power Differential Scale (VPD-total) (Birchwood *et al.* 2004) was used to measure the perceived power differential between voice and voice hearer, which includes the following constructs: power, strength, confidence, respect, ability to inflict harm, superiority and knowledge. This scale has good internal reliability (Cronbach's *α* = 0.85) with high 1-week re-test reliability (*r* = 0.82). The first item on the scale, the ‘pure’ power differential between voice and voice hearer, was analysed separately in keeping with our protocol and full trial. This item like the others in the scale is a bipolar construct on a five point scale (‘In relation to my voice I feel much more powerful….much less powerful’) and will be referred to as VPD-power.

#### Omniscience

The Personal Knowledge questionnaire/Omniscience scale (Birchwood *et al.* 2004) measured the voice hearer's beliefs about the voice's knowledge regarding personal information (e.g. ‘The voice knows everything about me and my past’).

#### Voices’ intentions

The Beliefs about Voices Questionnaire-Revised (BAVQ-R) (Chadwick *et al.* 2000) was used to assess key beliefs about voices’ omnipotence and intentions, whether benevolent or malevolent, as well as participants’ emotional and behavioural reactions towards their voices (resistance *v.* engagement). The scale has good test-retest (*r* = 0.89) and internal reliability (Cronbach's *α* = 0.85) and is widely used in hallucinations research.

### Auditory hallucinations

Voices were assessed using the Psychotic Symptoms Rating Scales (PSYRATS) (Haddock *et al.* 1999) and specifically the section about AH. The scale benefits from excellent psychometric properties with inter-rater reliability for the AH section ranging between 0.78 and 1.0.

### Depression, hopelessness & suicidal ideation

The Calgary Depression Rating Scale for Schizophrenia (Addington *et al.* 1993) is a widely used nine-item observer-rated measure specifically designed for schizophrenia, minimising contamination by negative symptoms and the extrapyramidal side effects of neuroleptics. The Beck Hopelessness Scale (Beck, 1988) was used to assess three aspects of hopelessness: feelings about the future, loss of motivation and expectations and the Beck Scale for Suicidal Ideation allows for a thorough examination of suicidal intent.

### Psychotic symptoms

The Positive and Negative Syndrome Scale (PANSS) (Kay *et al.* 1987) includes scales of positive symptoms, negative symptoms and general psychopathology and is used widely in schizophrenia research.

### Childhood neglect and trauma

The Childhood Trauma Questionnaire (Bernstein and Fink, 1988) is a widely used 28-item self-report inventory measuring retrospectively experiences of childhood abuse and neglect. It consists of five subscales: emotional abuse, physical abuse, sexual abuse, emotional neglect and physical neglect, reflecting, therefore, a broad range of early adverse experiences. The CTQ has proved to have high internal reliability with *α*-value ranging from 0.66 for the physical abuse subscale to 0.92 for the sexual abuse subscale. It has also demonstrated good test–retest reliability ranging from 0.79 to 0.86 for the five subscales over an average period of four months.

### Statistical methods

#### Baseline predictors of 18-month compliance

All statistical analyses were carried out using Stata version 13 (Stata Corporation, 2013). Thirty pre-randomisation, baseline predictor variables were considered including voice beliefs (13 variables), psychosis severity (four variables), parental abuse and neglect (five variables), demographic and historical factors (five variables) and effective functioning (three variables) in addition to treatment allocation and centre membership. In order to avoid confusion, readers should note that psychologists’ term ‘predictive variable’ does not have the same meaning as ‘predictive marker’ as used in the personalised medicine literature (Dunn *et al.* 2013). The latter is equivalent to the psychologists’ ‘moderator’ (a treatment effect modifier). Here, we are dealing with potential predictors that are assumed to have the same effect in both arms of the trial. The starting point (as in the initially reported evaluation of treatment efficacy (Birchwood *et al.* 2014) was a logistic regression model to predict 18-month full compliance by treatment allocation, centre membership (two binary dummy variables, C1 and C2) and baseline compliance on the VCS (with three levels). Centre membership was kept in all of the statistical models because of the trial design: randomisation taking place within (or stratified by) centre. Baseline compliance was not a statistically-significant predictor and was then dropped from the model. So, starting with a model containing the effects of treatment and centre membership, each of the 30 potential predictors were added in turn, and their predictive effects and associated statistical significance assessed using the likelihood ratio-based chi-square statistics. The effects of employment and marital status were not assessed since there was too little variation in these measures. The best (most statistically significant) predictor was kept in a new baseline model and each of the remaining 29 potential predictors again added in turn, and their predictive ability determined. This forward selection procedure was accompanied by a corresponding backward elimination, starting with a model with all potential predictors and proceeding until deletion of a potential predictor led to a statistically significant worsening in goodness-of-fit (again, indicated by the change in chi-square).

As a result of the above analyses, a selection of the potential predictors was made, based on their ability to predict outcome over and above treatment and centre membership. These baseline predictors were likely to be fairly highly correlated and their correlation matrix was then subjected to an exploratory principal components analysis, and the principal components extracted and saved to be used as potential predictors in their own right.

### Mediation analysis

Here, again, all initial statistical analyses were carried out using Stata version 13 (StataCorp, 2013). The analyses are aimed at evaluating the potential mediating role of voice power (VPD) (either the total score or the power differential item) in the treatment effect on compliance at 18 months. The main potential threat to the validity of the findings is confounding of the effect of the mediator (VPD) on outcome (Compliance), even though the treatment itself has been randomised. Another threat (or source of bias) is measurement error in the putative mediator. First, we concentrated on adjustment for confounding. The logistic regression model for the outcome (18-month compliance) initially included the effects of treatment and the following covariates: centre membership (two binary dummy variables, C1 and C2) the first principal component score (PCA1) obtained from the above analysis of potential predictors (defined in the ’Results’ section). This produced a revised intention-to-treat effect. PCA1 was included because it is likely to be an important confounder of the effect of the putative mediator on the clinical outcome – being a powerful predictor of both the VPD scores and 18-month compliance. Linear regression models for the putative mediator (VPD-total score at 9 months or the VPD-power item) included the effects of treatment and the same set of covariates. Finally, the second pair of logistic regression models for 18-month compliance was fitted, containing the same variables as the first ones above but, with the addition of the putative mediators: the 9-month values for either the VPD total score or the VPD power item. The latter models were also fitted using Stata's *paramed* command (Emsley *et al.* 2014; Dunn *et al.* 2015): in order to obtain estimates of the direct, indirect and total effects of treatment. Standard errors and 95% CI were used via the use of bias-corrected bootstrap methods (each based on 1000 bootstrapped samples) – see (Efron and Tibshirani, 1993). Readers should be aware that the total effects of treatment will not correspond exactly to the estimate reported in the original trial report (Birchwood *et al.* 2014). Hence, the phrase ‘revised intention-to-treat effect’ as used above. This is not a sign of disagreement but follows from the fact that treatment effects are being estimated using odds ratios, conditional on the other covariates in the model. As the additional covariates change so does the definition of the treatment effect (unlike the case when using linear regression/ANCOVA models for quantitative outcomes).

Finally, we evaluated mediation models in which 9- and 18-month measurements of the VPD-total score (or the VPD-power item) were assumed to be replicate measurements of an underlying latent variable (factor – labelled VPD-Total in the case of the total score, and VPD-Power for the power item). The latent factor was used to address measurement error. The replicate measurements for each of the two factors were assumed to have zero relative bias and the same measurement error variances (precision – i.e. they were assumed to be parallel measures). We would, of course, expect the precision of the power item to be considerably lower than that for the total score. This simple factor analysis model was then fitted simultaneously with a logistic regression model to predict 18-month compliance using the structural equations program Mplus version 7.0 (Muthen and Muthen, 2012). The covariates in the latter were treatment, centre membership (C1 and C2), PCA1 and the VPD factor as the putative mediator (either the VPDT or VPDP).

## Results

### Intention to treat analysis

The evaluation of the primary outcome was through an intention-to-treat (ITT) analysis using a logistic regression, allowing for centre membership and severity of command hallucinations at baseline. This is detailed in our primary publication (1) but in summary, 197 participants (see Table 1) were randomly assigned (98 to CTCH + TAU and 99 to TAU), representing 81.4% of eligible individuals. At 18 months, 46% of the TAU participants fully complied compared to 28% of those receiving CTCH + TAU (odds ratio = 0.45, 95% CI 0.23–0.88, *p* = 0.021). A summary of outcome and mediator measures over time by randomisation group is shown in Table 2. The estimate of the treatment effect common to both follow-up points was an odds ratio of 0.57 (95% CI 0.33–0.98, *p* = 0.042); that for 18-month compliance was an odds ratio of 0·45, 95% CI 0.23–0.88, *p* = 0·021. The total estimated treatment effect for VPD-total common to both time points was −1.819 (95% CI −3.457 to −0.181, *p* = 0.03). For the VPD-power differential item, the estimated treatment effect for both time-points was –0.52 (–0.849 to –0.185, *p* = 0.002).

**Table 1.** Description of COMMAND trial sample at baseline

	Total (%)
Gender (male):	113 (57%)
Site: Birmingham/Leicester	87 (44%)
Manchester	48 (24%)
London	62 (31%)
Ethnicity: non-white	68 (35%)
Employment: Employed	16 (32%)
Unemployed	144 (74%)
Other	19 (10%)
Cohabiting: yes	20 (10%)

**Table 2.** Summary of outcome and mediator measures over time by randomisation group

		Baseline	9 months	18 months
Full compliance	CTCH	84 (86%)	41 (48%)	22 (28%)
	TAU	81 (81%)	49 (55%)	39 (46%)
VPD power: mean (s.d.)	CTCH	3.9 (1.2) *n* = 97	2.8 (1.2) *n* = 87	2.8 (1.3) *n* = 76
	TAU	4.0 (1.0) *n* = 98	3.3 (1.4) *n* = 86	3.2 (1.4) *n* = 81
VPD total: mean (s.d.)	CTCH	26.5 (5.5) *n* = 95	21.3 (5.9) *n* = 87	22.4 (6.2) *n* = 75
	TAU	27.4 (4.6) *n* = 96	24.0 (6.4) *n* = 85	23.4 (6.9) *n* = 81

VPD, Voice power differential; CTCH, Cognitive therapy for command hallucinations; TAU, Treatment as usual.

### Baseline predictors of 18-month compliance

In addition to the effect of treatment there were also centre differences in 18-month compliance; however, once other baseline predictors were added to the model these centre effects were very variable. When each of the potential predictors (Table 3) were added to the logistic model, one at a time, 11 were found to have a statistically-significant effect, using the uncorrected nominal significance level of 0.05. The results are shown in Table 4, ranked according to their associated *p* value. The best predictor was BAVQ Omnipotence with stronger beliefs linked to compliance (*p* = 0.005). When the latter was kept in the model and the other potential predictors were then added, one at a time, the only one to significantly add to the prediction was CTQ emotional abuse (*p* = 0.045). Bearing in mind the repeated testing or multiplicity problem (i.e. family-wise error rates), the latter is almost certainly of no consequence. A backwards elimination confirmed the above results, yielding a model that contained treatment, centre and BAVQ Omnipotence.

**Table 3.** Baseline predictors: summary statistics

Variable	Observed	Mean	s.d.	Min	Max
Age at onset	178	22.140	10.562	5	63
PANSS Positive	197	19.381	4.865	10	33
PANSS Negative	195	16.021	6.293	7	34
PANSS General	195	36.374	8.602	17	71
PANSS Total	195	71.733	16.559	38	132
CDSS (Depression)	197	12.091	6.008	0	27
BHS (Hopelessness)	193	10.720	5.395	1	20
BSI (Suicidal thinking)	196	9.908	9.372	0	34
PKQ (Omniscience)	197	10.594	3.218	1	15
VPD (Voice power)	195	3.923	1.103	1	5
VPD total	191	26.953	5.054	9	35
PSYRATS total	194	32.629	4.400	18	41
BAVQ Malevolence	197	13.051	4.332	2	18
BAVQ Benevolence	196	3.367	3.989	0	15
BAVQ Omnipotence	197	13.487	3.748	0	18
BAVQ Resistance (total)	197	21.381	4.856	5	27
BAVQ Resistance (E)	197	9.853	2.302	1	12
BAVQ Resistance (B)	197	11.528	3.599	0	15
BAVQ Engagement (total)	197	5.061	4.919	0	21
BAVQ Engagement (E)	197	1.990	2.837	0	12
BAVQ Engagement (B)	197	3.071	2.874	0	12
CTQ (Emotional abuse)	192	14.135	6.677	5	25
CTQ (Physical abuse)	190	10.484	5.818	5	25
CTQ (Sexual abuse)	190	10.326	7.411	5	25
CTQ_(Emotional neglect)	192	12.995	6.437	5	25
CTQ (Physical neglect)	192	9.547	4.845	5	25

VPD, Voice power differential; BAVQ, Beliefs about voices questionaire; CTQ, Childhood trauma questionaire; PANSS, Positive and negative syndrome scale; BSI, Beck suicide inventory; BHS, Beck hopelessness scale; CDSS, Calgary depression scale for schizophrenia; PKQ, Personal knowledge questionaire; PSYRATS, Psychosis rating scales.

**Table 4.** Significant individual predictors of 18-month compliance

Predictor	Odds ratio	s.e.	*p* value	95% CI	
BAVQ Omnipotence	1.163	0.063	0.005	1.046	1.292
BAVQ Malevolence	1.122	0.051	0.011	1.027	1.226
CTQ (Physical abuse)	1.076	0.032	0.013	1.015	1.139
CTQ (Emotional abuse)	1.071	0.030	0.014	1.014	1.131
CDSS (Depression)	1.074	0.032	0.015	1.014	1.138
VPD Voice power (total)	1.099	0.043	0.017	1.017	1.188
BSI (Suicidal thinking)	1.046	0.020	0.018	1.008	1.085
PSYRATS (voices) total	1.105	0.047	0.019	1.017	1.201
CTQ_(Emotional neglect)	1.065	0.030	0.024	1.008	1.125
PKQ (Omniscience)	1.141	0.066	0.024	1.018	1.279
PANNS Positive	1.074	0.038	0.044	1.002	1.151

VPD, Voice power differential; BAVQ, Beliefs about voices questionaire; CTQ, Childhood trauma questionaire; PANSS, Positive and negative syndrome scale; BSI, Beck suicide inventory; CDSS, Calgary depression scale for schizophrenia; PKQ, Personal knowledge questionaire; PSYRATS, Psychosis rating scales.

In a further set of analyses, a principal component analysis was carried out on the correlation matrix for the eleven statistically significant predictors in Table 4. The first four principal components had eigenvalues of 3.690, 1.652, 1.137 and 1.034, respectively (explaining 68% of the total variation). Returning to the predictive models, the only principal component to predict 18-month compliance was the first one (which was essentially the average, after standardisation, of the eleven selected baseline variables). The estimated odds ratio for an increase of one unit of the 1^st^ principal component was 1.503 (s.e. 0.165; *p* value <0.001; 95% CI 1.211–1.865). The estimated odds ratio for the treatment effect in this model was 0.476 (s.e. 0.179; *p* value 0.048; 95% CI 0.228–0.993). Manchester had lower levels of 18-month compliance than Birmingham/Leicester (odds ratio 0.329; s.e. 0.156; *p* value 0.019; 95% CI 0.130–0.834), as did London (odds ratio 0.300; s.e. 0.135; *p* value 0.008; 95% CI 0.124–0.727). There was no suggestion that the treatment effect was in anyway moderated by the level of the baseline first principal component (i.e. the 1st principal component by treatment interaction had a *p* value of 0.915).

The eleven baseline predictor variables given equal weight in the PCA 1st principal component were: BAVQ Omnipotence and Malevolence scales, CTQ Physical abuse and Emotional abuse scales, CDSS (Depression), VPD Voice power (total), BSI (Suicidal thinking) PSYRATS voices (total), CTQ Emotional neglect, PKQ (Omniscience) and PANSS Positive total. These encompass beliefs about voices power and threat; childhood trauma; depression and self-harm all of which have featured as key features of our cognitive model of voices. We interpret this component therefore as the cognitive-affective dimension of the experience of hearing voices.

### Mediation analysis

It is hypothesised that CTCH will give the patient more power over the voice and the ability to resist its commands and so make them less likely to comply. The power differential between the voice and the patient was measured by the VPD total. The power item is a 1–5 rating where a score of one indicates power with the individual and 5 indicates that the power lies with the voice. An improvement would therefore be indicated by a reduction in the total VPD-total and VPD-power items. We are hypothesising that VPD power at 9 months mediates the impact of treatment on compliance at 18 months. These hypotheses are supported by the results provided below.

Results of fitting predictive models for 18-month compliance, and for 9-month VPD-total and VPD-power scores, are given in Table 5*a–c*. These simply confirm the original ITT analyses. Note that both centre membership and PCA1 are included in all models evaluating the effect of the mediator on outcome. The next step involves the inclusion of the putative mediators in the outcome model. These results are given in Table 5*d* and *e*. In Table 5*d*, it can be seen that the effect of the proposed mediator is highly statistically significant (although the estimated odds ratio is very close to unity, it should be remembered that this is the effect of increasing the 9-month VPD total by a single unit). The effect of treatment not explained by the 9-month VPD-total has an estimate (odds ratio 0.526) that is closer to the null than that given in Table 5*a*, again consistent with the hypothesis of mediation by the 9-month VPD-total measure. However the estimate of this direct effect is very imprecise (see 95% CI) and not very informative. The corresponding analysis, evaluating the possible mediating effect of 9-month VPD-power item indicates that there is little evidence of mediation by this measure (see Table 5*e*). We present what we believe is a more interpretable statistic in the following paragraph.

**Table 5.** Revised intention-to-treat (ITT) estimates, together with the effects of proposed mediators.

(a) 18-month compliance: ITT			
Covariate	Odds ratio	*p*-value	95% CI
Treatment	0.476	0.048	0.228–0.993
C1	0.329	0.019	0.130–0.834
C2	0.300	0.008	0.124–0.727
PCA1	1.503	<0.001	1.212–1.865
(b) 9-month VPD total: ITT			
Covariate	Coef.	*p*-value	95% CI
Treatment	−2.884	0.002	−4.720 to −1.048
C1	−2.040	0.079	−4.321 to 0.241
C2	−0.349	0.755	−2.552 to 1.855
PCA1	0.999	<0.001	0.505–1.493
(c) 9-month VPD power: ITT			
Covariate	Coef.	*p*-value	95% CI
Treatment	−0.545	0.006	−0.933 to −0.157
C1	−0.409	0.096	−0.891 to 0.073
C2	−0.143	0.544	−0.609 to 0.322
PCA1	0.131	0.014	0.027–0.236
(d) 18-month compliance with 9-month VPD total as a mediator			
Covariate	Odds ratio	*p*-value	95% CI
Treatment	0.526	0.116	0.236–1.173
9-m VPD total	1.098	0.009	1.023–1.179
C1	0.278	0.012	0.102–0.752
C2	0.213	0.002	0.080–0.571
PCA1	1.457	0.002	1.146–1.852
(e) 18-month compliance with 9-month VPD power as a mediator			
Covariate	Odds ratio	*p*-value	95% CI
Treatment	0.461	0.054	0.210–1.012
9-m VPD power	1.242	0.178	0.906–1.701
C1	0.260	0.007	0.097–0.698
C2	0.224	0.002	0.086–0.583
PCA1	1.524	<0.001	1.210–1.921

PCA1, Principal component 1; VPD, Voice power differential.

Stata's *paramed* was used to estimate the direct and indirect effects of treatment, based on the same models for the putative mediator and final outcome as described above. Here, *paramed* produces three estimates: the total effect of treatment, the natural indirect effect of treatment and the natural direct effect of treatment, together with their 95% CI. The total effect (te) is equivalent to the above ITT estimate. The natural indirect effect (nie) is defined as the effect on 18-month compliance of changing the value of the mediator (VPD) from that which it would have had in the control condition to that which it has under treatment, whilst setting Treatment = 1 (i.e. *everyone* receives the intervention). The corresponding natural direct effect (nde) is the effect of receiving treatment (changing Treatment from 0 to 1) whilst fixing the mediator at the value it would have had under the control condition (Treatment = 0). Using 9-month VPD total as the mediator, we obtain the following estimates: te = 0.402 (95% CI 0.153–0.998); nie = 0.763 (95% CI 0.456–0.939); nde = 0.526 (95% CI 0.215–1.503). Using 9-month VPD power we obtain: te = 0.410 (95% CI 0.166–1.107); nie = 0.889 (95% CI 0.603–1.005); nde = 0.461 (95% CI 0.191–1.193). Note that the estimated direct effects are exactly the same as those reported in Table 5*d* and *e*, respectively, but with wider confidence intervals. The estimated natural indirect effects (both odds ratios less than unity) correspond to that component of the treatment effect that is fully explained by its effect on the mediator. We conclude that 9-month VPD total is a mediator of the effect of treatment on 18-month compliance, but it is difficult to establish whether all of the treatment effects is explained by it (the fact that the corresponding direct effect is not significantly different from unity does not mean that it actually is unity – we cannot rule out lower values). The estimated natural indirect effect for 9-month VPD power, however, is not statistically significantly different from unity (the 95% CI straddles the value of 1) and conclude that we have not been able to demonstrate that VPD power is a mediator.

Finally, the latent variable model evaluating the mediating effect of VPD-total factor led to an estimated effect of the mediator on the outcome of 1.113 (95% CI 0.995–1.245), consistent with the estimate in Table 5*d*, but less precise. Measurement error does not appear to be a major source of bias, here. The corresponding result for the VPD power factor was 1.381 (95% CI 0.829–2.303). Again, measurement error does not appear to be a major source of bias, but the estimates are so imprecise that we stick with the conclusion that we have not been able to demonstrate that VPD power is a mediator of the treatment effect.

## Discussion

The COMMAND trial tested the effectiveness of our targeted CBT to reduce the risk of harmful compliance with commanding voices in those who had complied within the previous nine months. The trial showed a large and significant reduction in both the compliance and the central target of the therapy, beliefs about the power of voices, in particular, the difference in power between voice and voice hearer. In this paper, we addressed two central and related questions that are at the heart of the cognitive model and the CBT: to what degree does voice power and omnipotence predict compliance at final follow-up and did it act as the mediator of change? The logistical regression procedure identified eleven baseline predictors of outcome, over and above treatment and centre membership. The stepwise elimination routine identified BAVQ voice omnipotence as the best individual predictor; the other baseline predictors did not seem to have any predictive ability not explained by BAVQ omnipotence. These baseline predictors were however highly correlated and their correlation matrix was then subjected to an exploratory principal components analysis; the principal components extracted were saved to be used as potential predictors in their own right. The eleven predictors loaded equally on the first principal component, comprising beliefs about voices power and threat; childhood trauma; depression, hopelessness and suicidal thinking all of which are the key features of our cognitive model of voices. We describe this as the cognitive-affective dimension of voice hearing and it is this that strongly predicted 18-month compliance. In testing the mediating effect of voice power differential, the first principal component was an important variable to include as a confounder in the mediation model, confirmed by its highly significant prediction of 9-month compliance.

In the subsequent mediation analysis, controlling for any confounding effect of centre and PCA1, we found that the estimated natural indirect effects of treatment corresponded to that component of the treatment effect that is fully explained by its effect on the hypothesised mediator: 9-month voice power differential. There were no direct effects of treatment once the effect on the mediator was accounted for. We conclude that 9-month VPD-total was a mediator of the effect of treatment on 18-month compliance. While we cannot rule out a direct effect of treatment on outcome, it is very difficult to obtain a precise estimate of this residual (unmediated) treatment effect; if there were comparable trials it would be an ideal candidate for an IPD (individual patient data) meta-analysis.

We have been able to demonstrate the mediating role of VPD-total, but have failed to demonstrate the same for VPD power item. This may be a sample size (statistical power) problem arising from a measure that contains considerable error variance. We cannot rule out a direct effect of treatment on outcome, and it is very difficult to obtain a precise estimate of this residual (unmediated) treatment effect.

### Strengths and limitations

This trial mediation analysis has many strengths that lend confidence to the findings. This was a carefully designed and implemented RCT specifically targeted on voice power to achieve change in compliance with command hallucinations, and with a corresponding a priori hypothesis that voice power was the mediator. We employed a large sample size of a complex and difficult to recruit group, with a high rate of follow-up in both groups (83%). We made a careful search and allowance for predictors that could be used for confounder adjustment in the mediation models. Similarly, a careful check was made for the sensitivity of the results to measurement errors in the mediators. The prediction analysis was based on a prior hypothesis based on our cognitive model and on evidence from a preliminary study showing that voice power was the principal predictor of 9-month compliance (Bucci *et al.* 2013).

There are some caveats. First, although the data support the view that the effect of treatment on compliance is mediated by voice power differential (VPD-total) there is insufficient power to confidently rule out any possibility of some effect of treatment not mediated in this way (i.e. we cannot exclude a direct effect of treatment, including non-specific on compliance). Second, the trial did not find a differential impact of treatment on distress linked to voices (distress declined in both groups). Our previous cross-sectional and prospective work had led us to predict a treatment effect on voice-related distress, but this was not found, meaning we were unable to conduct a mediation analysis on this variable (though distress was a secondary outcome). Finally, there may be other methods of enhancing the power of the voice hearer: ‘avatar therapy’ (Leff *et al.* 2013) is rooted in our cognitive model and uses facsimile of voices to develop control and enhance power; similarly ‘relating’ therapy (Hayward *et al.* 2016) involves the rehearsal of assertive approaches to relating to the voice and to another significant problematic relationship.

We can conclude that the treatment effect in this trial was mediated by voice power but we cannot rule out the influence of other, for example, non-specific effects of the therapy, that could account for its impact.

### Implications

This trial mediation analysis brings to full circle our influential cognitive model we formulated over 20 years ago (Chadwick & Birchwood, 1994). We argue that it provides convincing evidence that in relation to harmful behaviour at least, the perceived power of voices to threaten the individual, relative to the perceived power of the individual to challenge and mitigate this threat (power differential), is a strong and malleable influence on voice-related behaviour. Had the mediation analysis not supported the role of voice power, this would have questioned the foundation of the therapy in its focus on voice power differential and raised questions about other active agents, for example, demand characteristics of the trial, a placebo effect or bias. On the contrary, the mediation analysis supports the clinical model and opens the door for further research to understand the development of these appraisals as an interpersonal phenomenon and to explore the clinical model and its applicability to the generality of distressed voice hearers and to other symptoms where these appraisals are active, particularly persecutory delusions. Research is needed to augment this approach with methods to further reduce distress; in our structural equation model of voice distress (Birchwood *et al.* 2004) we identified that generalised subordination to others and depression appeared to be powerful drivers of distress in the relationship with voices and these may be targets for intervention for lasting change in voice distress. For the clinician working with individuals with harmful voices, assessment should include the perceived power of the voice relative to the individual, the sources of voice power and those of the voice hearer. We have documented the majority of cases (Byrne *et al.* 2006; Meaden *et al.* 2013) in the trial and pilot for the interested clinician. We have argued (Birchwood, 2015) that the next generation of CBTp should focus not on complex packages of care, where the mechanisms are unclear and the ability of the intervention to modify them is lost in the scattershot; rather we offer this paradigm of theoretical development, leading to targeted intervention focused on the mechanism of interest, as the path most likely to maximise clinical effectiveness of the next generation of cognitive therapy for psychosis.
